# Amino Acid Substitutions in Bacteriocin Lactolisterin BU Reveal Functional Domains Involved in Biological Activity Against *Staphylococcus aureus*

**DOI:** 10.3390/molecules30153134

**Published:** 2025-07-26

**Authors:** Lazar Gardijan, Milka Malešević, Miroslav Dinić, Aleksandar Pavić, Nikola Plačkić, Goran Jovanović, Milan Kojić

**Affiliations:** 1 Institute of Molecular Genetics and Genetic Engineering, University of Belgrade, 11000 Belgrade, Serbia; lazar.gardijan@imgge.bg.ac.rs (L.G.); milka.malesevic@imgge.bg.ac.rs (M.M.); miroslav.dinic@imgge.bg.ac.rs (M.D.); aleksandar.pavic@imgge.bg.ac.rs (A.P.); nikola.plackic@imgge.bg.ac.rs (N.P.); 2 Institute of Virology, Vaccines and Sera “Torlak”, 11000 Belgrade, Serbia

**Keywords:** lactolisterin LBU, antimicrobial peptide, bacteriocin, amino acid substitution, *Staphylococcus aureus*, AlphaFold, zebrafish toxicity, virulence

## Abstract

The emergence of multidrug-resistant pathogens has driven the development of novel antimicrobial peptides (AMPs) as therapeutic alternatives. Lactolisterin LBU (LBU) is a bacteriocin with promising activity against Gram-positive bacteria, including *Staphylococcus aureus*. In this study, we designed and evaluated a panel of amino acid variants of LBU to investigate domain–activity relationships and improve activity. Peptides were commercially synthesized, and their effect was evaluated for minimal inhibitory concentration (MIC), minimal bactericidal concentration (MBC), hemolytic activity, cytotoxicity, in vivo toxicity, and virulence modulation. AlphaFold3 structural prediction of LBU revealed a four-helix topology with amphipathic and hydrophobic segments. Helical wheel projections identified helices I and IV as amphipathic, suggesting their potential involvement in membrane interaction and activity. Glycine-to-alanine substitutions at helix I markedly increased antimicrobial activity but altered toxicity profiles. In contrast, changes at helix junctions and kinks reduced antimicrobial activity. We also showed differential regulation of virulence genes upon sub-MIC treatment. Overall, rational substitution enabled identification of residues critical for activity and toxicity, providing insights into therapeutic tuning of lactolisterin-based peptides.

## 1. Introduction

Antimicrobial resistance (AMR) is recognized as one of the most pressing public health threats of the 21st century, with *Staphylococcus aureus* ranking among the high-priority pathogens responsible for life-threatening skin, soft tissue, and bloodstream infections worldwide [1]. In 2021, methicillin-resistant *S. aureus* (MRSA) accounted for an estimated 550,000 associated deaths and 130,000 deaths directly attributable to resistance—an increase of more than double since 1990 [2]. Forecasts suggest that without significant intervention, global deaths attributed to antimicrobial resistance (AMR) could rise to 1.91 million by 2050, with older adults being the most affected group [2]. Therapeutic failure, prolonged hospitalization, and an escalating socio-economic burden underscore the urgent need for new anti-staphylococcal agents that bypass canonical antibiotic targets and minimize cross-resistance [3,4].

Antimicrobial peptides (AMPs) are promising alternatives or adjuncts to conventional antibiotics. This broad class encompasses peptides derived from all domains of life, unified by their primary function: antimicrobial activity. Since they can be found in every organism, their synthesis, structure, and function are diverse—ranging from small unmodified peptides to two peptides, cyclic peptides, and heavily modified peptides [5,6]. Bacteriocins—ribosomally synthesized antimicrobial peptides produced by bacteria—have gained attention as next-generation antimicrobials because of their narrow spectra, rapid mode of action, and low propensity for inducing resistance [7,8]. Although promising antibiotic alternatives, few bacteriocins are in commercial use as food preservatives, including nisin [9], pediocin PA1 [10], and Micocin^®^ [11]. While no bacteriocins have been approved for human use to date, several are currently undergoing clinical trials [12,13]. Bacteriocins are classified based on their structure, mode of action, and producing organism [7]. They are typically grouped into classes, such as class I (lantibiotics, containing unusual amino acids), class II (small, heat-stable peptides), class III (large, heat-labile proteins), and class IV (complex bacteriocins containing lipid or carbohydrate moieties) [7]. Small, post-translationally unmodified leaderless class II bacteriocins are particularly attractive candidates: they are active immediately after translation, display potent activity against Gram-positive pathogens, and can be relatively easily bio-engineered [14,15]. Although many bacteriocins have been discovered and characterized, several obstacles—cytotoxicity, serum instability, limited recombinant expression, and incomplete understanding of structure–function relationships—still hamper commercialization and clinical translation [13,16].

LBU is a 43-residue leaderless class IId bacteriocin first isolated from *Lactococcus lactis* subsp. *lactis* bv. diacetylactis BGBU1-4 and subsequently shown to inhibit a broad panel of Gram-positive genera, including *Listeria*, *Staphylococcus*, *Bacillus*, enterococci, and streptococci [17]. It is thermostable, shows activity in a wide pH range, and is salt-tolerable but sensitive to gut peptidases [17]. It belongs to the IId group—unmodified leaderless bacteriocin—and it shares sequence homology with aureocin A53, aureocin A53-like peptide, lacticins Q and Z, and BHT-B, with whom LBU shares ~63% sequence similarity [17]. Genome mining of *L*. *lactis* BGBU1-4 uncovered many putative bacteriocin genes, yet only LBU (5.161 kDa) and a naturally occurring truncated form (LBU1-31, 3.642 kDa) are produced [18]. Systematic deletions showed that the first 24 amino acids are essential for antimicrobial activity, whereas residues 25–43 modulate bacterial species selectivity [18]. It showed promising results in pilot food application studies—including pork refrigerated preservation [19] and using LBU producer as a soft-cheese adjunct [20]. Safety studies showed no signs of systemic inflammation in mice fed cheese enriched with the LBU-producing strain, although a transient cytokine response was observed in gut lymph nodes [20]. Recombinant LBU exhibited low cytotoxicity and induced dose-dependent hemolysis [19]. Despite the use of various expression vectors and host systems—including *Lactococcus*, *E. coli*, and *Pichia pastoris*—as well as tag-sandwich strategies (unpublished data), all our attempts to express LBU were unsuccessful. It should be noted that Dong et al. [19] successfully expressed LBU using the *P. pastoris* expression system. Due to the insufficient yield of LBU obtained through heterologous expression, we opted for chemical synthesis as an alternative approach to obtain active peptide material. This strategy has also been employed by other researchers facing similar challenges with low bacteriocin production, highlighting chemical peptide synthesis as a practical solution for obtaining larger quantities of functional compounds [21,22,23].

Although initial studies identified amphipathic α-helical segments and a cationic surface as key determinants of activity [17,18], the precise contribution of individual amino acid residues to antimicrobial potency, membrane selectivity, and host cell compatibility remains unresolved. Importantly, to the best of our knowledge, no systematic mutational analysis of LBU has been reported. To some extent, the effects of amino acid substitutions on the antimicrobial activity and toxicity profile of a bacteriocin can be predicted; however, experimental validation remains essential. For example, increasing the polar residue content may enhance solubility in aqueous environments but could disrupt amphipathic properties [24,25]. Similarly, altering the number or position of charged residues may impair initial membrane interactions while potentially improving the hydrophilic face of pores in pore-forming peptides [26,27]. To bridge this gap, we performed an amino acid mutational scan and selected substitutions at key positions to probe the structure–activity landscape of LBU. This approach yielded three single-point variants, G3A, G7A, and G13A, that exhibited a lower minimum inhibitory concentration (MIC) against *S. aureus* ATCC 25923 than the native LBU while retaining its physicochemical profile.

Despite the demonstrated antimicrobial potential and early food application studies involving LBU, a number of key questions remain unanswered. Most notably, there has been no systematic investigation into how specific amino acid residues contribute to its antimicrobial potency, membrane selectivity, and host toxicity. In addition, efforts to produce LBU recombinantly have been largely unsuccessful, limiting the scope of functional and mechanistic studies. As such, the structure–activity relationship of LBU remains poorly defined, and its therapeutic potential has not been fully explored.

In this study, we leveraged molecular, microbiological, and physicochemical end points, together with in vitro and in vivo toxicity analysis, to unlock the therapeutic potential of more active variants relative to LBU while simultaneously assessing the importance of single amino acids in structure–activity relationships through amino acid mutational scans. In addition, using RT-qPCR, we assessed the effect of the beforementioned variants on the virulence-associated gene expression of *S. aureus* ATCC 25923, both in rich media and a host-mimicking environment.

## 2. Results

### 2.1. Lactolisterin LBU Structure Prediction and Variant Design

**Figure 1:**
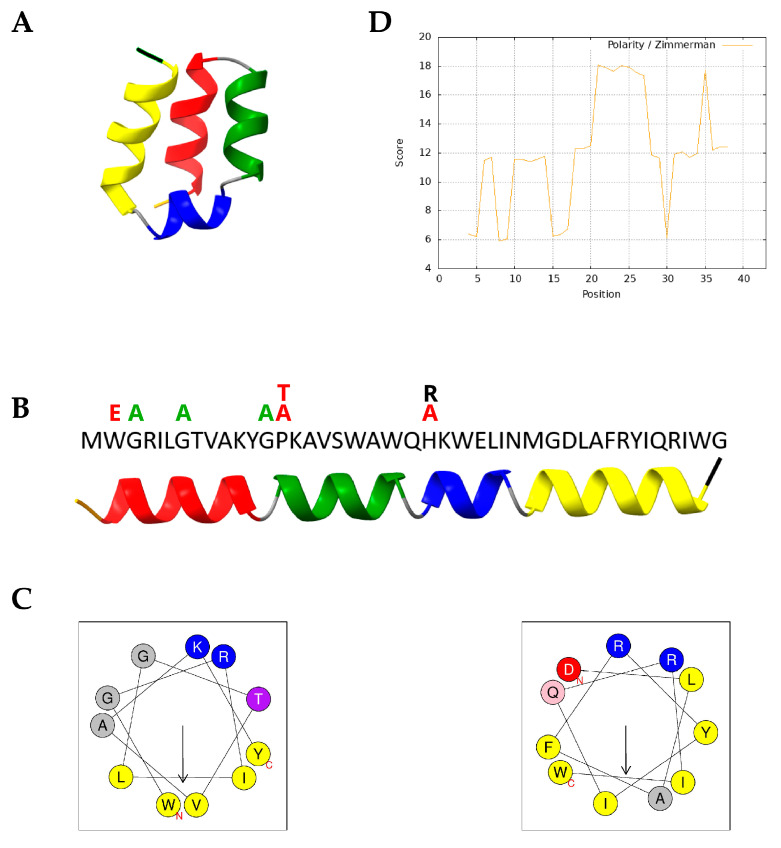
Structural and biophysical characterization of lactolisterin LBU (LBU). (**A**) AlphaFold-predicted 3D structure of LBU, highlighting the four-helix bundle architecture. (**B**) LBU amino acid sequence aligned with the relaxed 3D prediction; secondary structure regions are color-coded. Amino acids above the sequence represent variants, and their colors represent antimicrobial activity change related to LBU: green—increased activity, black—none to small change, and red—decreased activity. (**C**) Helical wheel projections of the first (I, left) and fourth (IV, right) helices, visualizing residue properties: yellow—nonpolar, purple—polar uncharged, blue—positively charged, red—negatively charged, and grey—unclassified/other. Arrows indicate the direction and magnitude of the hydrophobic moment; projections for helices II and III are not included due to their short length. (**D**) Polarity plot of the LBU sequence using the Zimmerman polarity scale. Color scheme for panels (**A**) and (**B**): red—first helix (residues 2–12), green—second helix (14–22), blue—third helix (24–29), yellow—fourth helix (31–42), orange—N-terminus, black—C-terminus, and grey—unstructured/coil regions.

To investigate the structural features underlying the bioactivity of LBU, we utilized the AlphaFold Protein Structure Database [28] to retrieve a high-confidence predicted structure of the peptide. The model was visualized using UCSF ChimeraX 1.9 [29]. As shown in Figure 1A, the prediction shows that peptide adopts a compact, helix–turn–helix–turn–helix conformation.

In Figure 1B, the same structure is mapped onto the primary sequence, with a clear correspondence between helical domains and defined regions in the amino acid sequence.

This arrangement suggests a helical bundle stabilized by intramolecular contacts and possibly forming amphipathic surfaces for membrane interaction.

To quantify amphipathic potential, we used helical wheel projections and hydrophobic moment (μH) calculations using HeliQuest (Figure 1C). The results revealed a high hydrophobicity and hydrophobic moment, as well as positive net charge (z) for both helix I (H = 0.591, μH = 0.705, z = +2) and helix IV (H = 0.691, μH = 0.602, z = +1), confirming that these two segments form strongly amphipathic helices with spatially segregated hydrophobic and hydrophilic faces—typical of membrane-active antimicrobial peptides [30]. These helices are likely to mediate insertion into bacterial membranes and initial surface disruption. The polarity graph using the Zimmerman scale also supported this, which can be seen as alternating peaks and valleys of polar and nonpolar residues (Figure 1D).

In contrast, the area that includes helices II and III has low hydrophobic moment but high average hydrophobicity (H = 0.695, μH = 0.251, z = 0) and high polarity content, indicating a role in helix–helix packing or core stabilization and/or membrane-anchoring [31].

Taken together, these analyses suggest that helices I and IV are key drivers of membrane permeabilization and antimicrobial activity, while helices II and III contribute to the structural integrity and membrane-anchoring of LBU.

Based on this analysis, we selected potentially relevant residues as targets for substitution with the intention to change the amphipathic balance, charge, potential to modulate helical stability or membrane interaction, or, most obviously, alter the conserved regions in homologous peptides, such as BHT-B (Table 1). These variants were then probed for antimicrobial activity; those with elevated activity, compared to LBU, were further functionally analyzed.

1. Helix I stability variants (G3A, G7A, W2E).

Helix I (residues 2–12) probably anchors LBU to bacterial membranes and contributes the bulk of the peptide’s amphipathic moment. Gly at positions 3 and 7 reduces intrinsic helicity yet preserves flexibility essential for membrane insertion. Replacing these Gly with alanine (G3A, G7A) increases helical propensity without altering net charge, allowing us to test whether tighter helix packing enhances antimicrobial potency or, instead, aggravates cytotoxicity. The W2E mutant exchanges the membrane-anchoring Trp for a negatively charged Glu, disrupting both hydrophobic surface area and aromatic membrane interactions. This variant sets a lower boundary for hydrophobicity required to maintain activity while simultaneously probing the role of N-terminal charge distribution.

2. Pro-kink and its neighborhood (G13A, P14T, P14A).

Proline introduces a bend, producing a helix–turn–helix motif that spatially separates two amphipathic segments. G13A (adjacent to the kink) tests whether local backbone flexibility upstream of Pro modulates the bend angle and, by extension, lipid packing defects exploited during membrane translocation. P14T preserves the side-chain polarity of proline while restoring backbone hydrogen-bonding capacity, offering a subtle way to relax the kink without deleting it outright. Additionally, this amino acid substitution is seen in the LBU homolog, BHT-B bacteriocin. P14A eliminates the rigid imide ring, creating an extended continuous helix; this provides a maximal contrast to the native hinge and allows us to evaluate whether a straightened backbone improves peptide insertion.

3. Histidine functionality (H23A, H23R).

His23 occupies the link between two amphipathic helices and is predicted to participate in pH-dependent membrane disruption or dimerization. Substituting His with Ala (H23A) removes both the imidazole side chain and its titratable positive charge, clarifying the importance of His electrostatic interactions. Conversely, replacement with Arg (H23R) retains a permanent positive charge while abolishing the pH switch, permitting us to separate charge effects from protonation dynamics and to probe whether a stronger cation enhances bacterial binding at the expense of host cell compatibility.

4. Double mutant (G3A/H23A).

Finally, the G3A/H23A double mutant combines enhanced N-terminal helicity with loss of the electrostatic handle, enabling a test for functional coupling between helix I stability and link surface chemistry. Any non-additive changes in antimicrobial activity will pinpoint synergistic (or antagonistic) interactions between these two structural domains.

5. BHT-B (BHT-B, N23H).

We also included BHT-B and its variant, BHT-B_N23H, a bacteriocin with the highest amino acid similarity to LBU [17]. The primary physicochemical differences between BHT-B and LBU lie at position 14, where BHT-B contains Thr and LBU contains Pro, and at position 23, where Asn in BHT-B is substituted by His in LBU. To explore structure–function relationships, specific residues in LBU were substituted to mimic those found in BHT-B. Conversely, the BHT-B_N23H variant features a targeted substitution designed to reflect the corresponding residue present in LBU.

By systematically modulating helical propensity, bend geometry, and surface charge, this variant set allows us to (i) map critical determinants of LBU’s antibacterial efficacy, (ii) identify sequence hotspots that drive host toxicity, and (iii) guide the rational optimization of next-generation LBU analogues with an improved therapeutic window.

### 2.2. Antimicrobial Activity Assessment

#### 2.2.1. Spot-on-the-Lawn Antimicrobial Testing

The antimicrobial activity screening assay of twelve chemically synthesized peptides was evaluated using a spot-on-the-lawn assay against *S. aureus* ATCC 25923. Each peptide was tested at serially decreasing concentrations (1000, 500, 250, 125, and 62.5 µg/mL) by spotting 5 µL aliquots onto freshly inoculated agar lawns.

**Figure 2:**
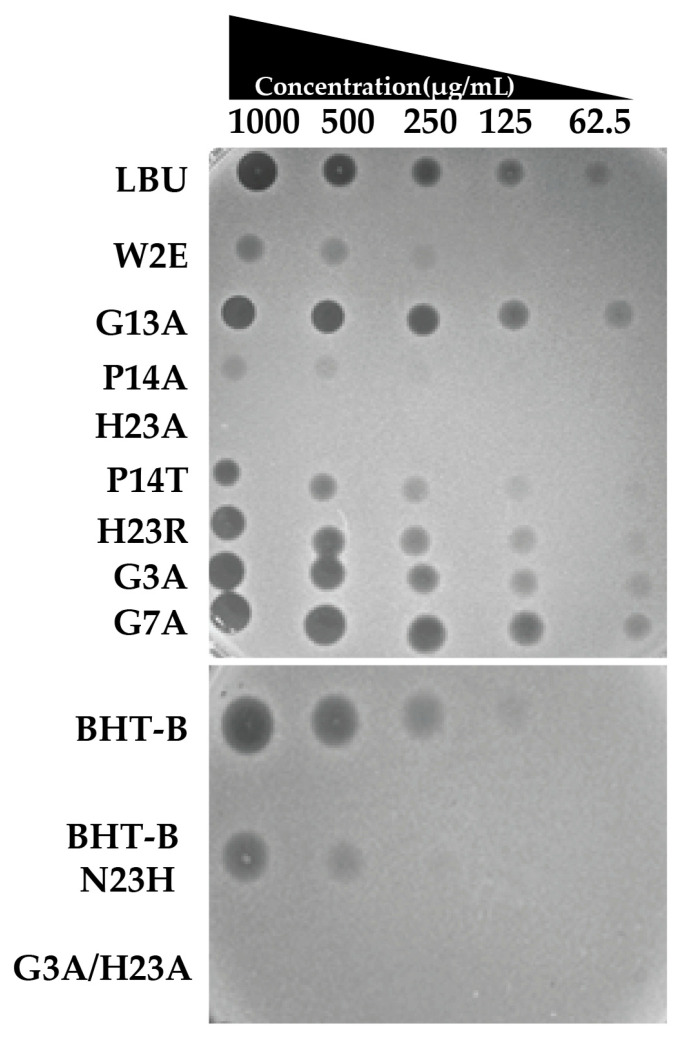
Spot-on-the-lawn assay of synthetic LBU and its peptide variants against *S. aureus* ATCC 25923.

The reference peptide LBU exhibited clear zones of inhibition across the tested concentrations, except 62.5 µg/mL. Among the variants, H23A and the double mutant G3A/H23A demonstrated complete loss of antimicrobial activity, with no observable inhibition at any concentration. P14A displayed severely reduced activity, producing only faint inhibition zones at the highest concentrations (1000 and 500 µg/mL), whereas P14T retained partial activity with a mild decrease in zone diameter compared to LBU (Figure 2).

The W2E variant also showed decreased antimicrobial potency, producing smaller inhibition zones, especially at lower concentrations. In contrast, H23R, G3A, G7A, and G13A maintained antimicrobial activity comparable to the wild-type LBU peptide across all concentrations tested, indicating that these substitutions did not impair function under the tested conditions.

BHT-B exhibited clear inhibition zones at concentrations down to 250 µg/mL, with a faint zone still visible at 125 µg/mL, indicating strong antimicrobial activity. In contrast, its variant BHT-B N23H showed inhibition only at 1000 and 500 µg/mL, suggesting markedly reduced potency.

These findings highlight that certain substitutions, such as H23A and the double mutation G3A/H23A, abolish activity, while others (e.g., G3A, G7A, H23R) preserve the antimicrobial profile of the parental peptide.

#### 2.2.2. MIC and MBC

The minimal inhibitory concentration (MIC) and minimal bactericidal concentration (MBC) of the parental peptide LBU and its amino acid variants were assessed against *S. aureus* ATCC 25923 (Table 2).

LBU exhibited an MIC of 12.5 µg/mL and an MBC of 25 µg/mL. Substitutions at the Gly residue (positions 3, 7, and 13) with Ala (G3A, G7A, and G13A) significantly enhanced antimicrobial potency, reducing the MIC to 6.25 µg/mL while maintaining the same MBC (25 µg/mL), except for G13A, where the MBC was lowered down to 12.5 µg/mL.

In contrast, variants with Pro substitutions (P14A and P14T) showed diminished efficacy (MIC = 25 µg/mL) and incomplete bactericidal activity, with MBC values exceeding 25 µg/mL and 50 µg/mL, respectively.

The H23R variant retained an MIC comparable to LBU (12.5 µg/mL) but displayed increased MBC (50 µg/mL), indicating a potential reduction in killing efficiency.

Notably, peptides with W2E, H23A, and double-substitution G3A/H23A failed to achieve growth inhibition at concentrations up to 50 µg/mL, highlighting a reduction in antimicrobial activity.

BHT-B exhibited the same MIC and MBC values as LBU, indicating comparable antimicrobial potency. In contrast, the BHT-B_N23H variant showed increased MIC (50 µg/mL and MBC (>50 µg/mL) values, which were higher compared to LBU with His at position 23. This highlights not only the functional importance of position 23, but also the influence of amino acid context on antimicrobial activity.

These results demonstrate that single Gly-to-Ala substitutions can enhance antimicrobial efficacy, whereas substitutions involving charged or bulky residues may be detrimental. Moreover, the structural context in which the substitution occurs also plays a critical role in determining its functional impact.

Based on their lower MICs relative to LBU, the variants G3A, G7A, and G13A were selected for further toxicological assessment and virulence modulation studies.

### 2.3. Safety Assessment

#### 2.3.1. Hemolysis

**Figure 3:**
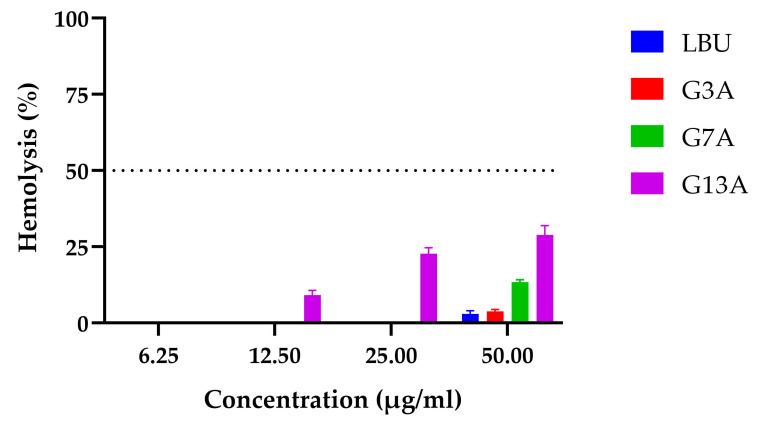
Hemolytic activity (%) of LBU and its Gly-to-Ala variants (G3A, G7A, G13A) on sheep erythrocytes at different peptide concentrations. The dotted line at 50% represents the HC_50_ threshold. Results are reported as mean ± standard deviation.

Hemolysis assays were performed on sheep erythrocytes to evaluate the hemolytic effect of LBU and its Gly-to-Ala variants (G3A, G7A, and G13A) at concentrations ranging from 6.25 to 50 µg/mL (Figure 3).

The results demonstrate that Gly-to-Ala substitutions at distinct positions in the peptide sequence differentially affect hemocompatibility, with G13A posing the greatest risk for hemolysis at antimicrobial concentrations.

The parental peptide LBU exhibited negligible hemolytic activity across all concentrations, with only 2.98% lysis observed at the highest dose (50 µg/mL). The G3A variant showed a similarly favorable profile, with no measurable hemolysis up to 25 µg/mL, and the G7A variant showed some membrane-disruptive potential at elevated concentrations (50 µg/mL)

In contrast, G13A induced dose-dependent hemolysis, starting at 12.5 µg/mL (9.12%) and rising sharply to 22.69% at 25 µg/mL and 28.86% at 50 µg/mL. This indicates a substantially greater erythrocyte-lytic capacity relative to LBU and the other variants.

As no peptide reached 50% hemolytic activity within the tested range, HC_50_ values could not be calculated, indicating a favorable erythrocyte safety profile.

#### 2.3.2. Cytotoxicity Toward Caco-2 Cells

The LBU and three Gly-to-Ala variants were evaluated for concentration-dependent cytotoxicity in Caco-2 monolayers using the LDH-release assay. Caco-2 cells were used in the toxicity assay to model the human intestinal epithelium, as they closely mimic the morphology and function of enterocytes. This makes them a relevant in vitro system for evaluating the potential cytotoxic effects of bacteriocins upon oral exposure or gastrointestinal application.

**Figure 4:**
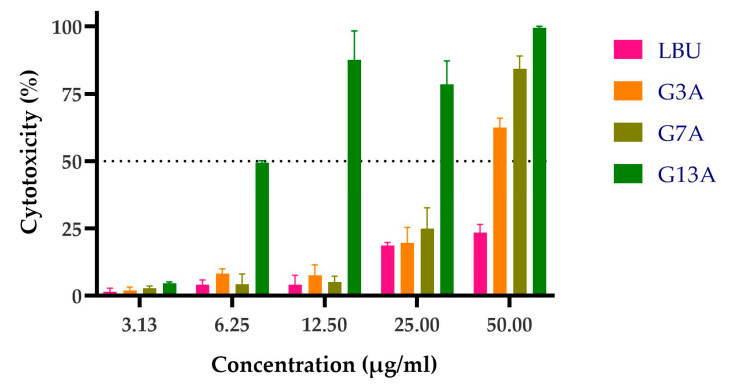
LDH cytotoxicity assay of Caco-2 cells at increasing concentrations. LBU and its variants (G3A, G7A, G13A) were tested in a standard LDH-release assay on Caco-2 cells. Values are expressed as % cytotoxicity (mean ± SD). The dotted line at 50% represents the LC_50_ threshold. Results are reported as mean ± standard deviation.

The results indicate a concentration-dependent increase in LDH release across all peptides. Importantly, LBU, G3A, and G7A remained non-cytotoxic up to 12.5 µg/mL, suggesting safe usage within this range for intestinal epithelial applications. In contrast, G13A displayed high cytotoxicity starting from 6.25 µg/mL, significantly limiting its therapeutic use (Figure 4).

**Figure 5:**
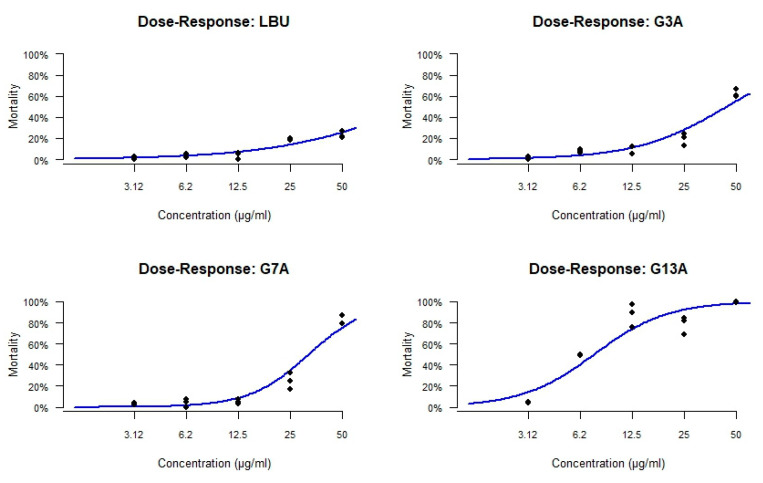
Dose–response curves of LDH release from Caco-2 cells after 24 h exposure to LBU and its Gly-to-Ala variants (G3A, G7A, G13A). Curves were fitted using nonlinear regression (variable slope, four-parameter logistic model); data points represent mean ± standard deviation (*n* = 3).

The dose–response curves (Figure 5) revealed distinct toxicity profiles for each peptide. The parental peptide LBU showed minimal toxicity, with no significant cytotoxicity even at the highest concentration tested (50 µg/mL), indicating an LC_50_ above 50 µg/mL. The G3A variant and the G7A variant demonstrated moderate toxicity, with calculated LC_50_ values of 43.91 ± 2.47 µg/mL and 31.93 ± 1.07 µg/mL, respectively. In contrast, the G13A variant exhibited pronounced embryo lethality in a dose-dependent manner, with an LC_50_ of 7.49 ± 0.26 µg/mL, highlighting a substantial increase in toxicity relative to the other variants.

The therapeutic index (TI), calculated as the ratio of LC_50_ to MIC, provided insight into the selectivity of each peptide toward bacterial cells versus host toxicity (Table 3). LBU did not exhibit 50% cytotoxicity at the tested concentration, so the TI was approximated to >4. G3A and G7A retained favorable safety profiles, with TIs of 7.03 and 5.11, respectively. However, G13A displayed a markedly reduced TI of 1.20, indicating limited therapeutic potential due to high host toxicity near its antimicrobial threshold.

#### 2.3.3. In Vivo Toxicity Assessment

To evaluate the in vivo toxicity of LBU and its variants, a dose–response effect on survival and inner organ development of zebrafish embryos was examined. Embryos were exposed to six different increasing concentrations of each peptide for 5 consecutive days (120 hfp) and examined for survival and signs of toxicity, following toxicological endpoints (Table S1). Based on the data obtained, the lethal concentration required to cause 50% mortality of the exposed embryos (LC_50_) was determined for each peptide using nonlinear regression analysis.

Data obtained in this assay showed that single amino acid substitutions in the LBU peptide significantly altered its toxicity profile in vivo. In particular, the G13A variant markedly increased embryotoxicity in *D. rerio*, raising concerns about the safety of this modification despite potential improvements in antimicrobial potency.

The reference peptide LBU exhibited an LC_50_ value of 6.53 µg/mL, while its G3A variant demonstrated slightly reduced toxicity, with an LC_50_ of 5.69 µg/mL. In contrast, the G7A and G13A mutants showed substantially increased toxicity, with LC_50_ values of 2.98 µg/mL and 1.56 µg/mL.

**Figure 6:**
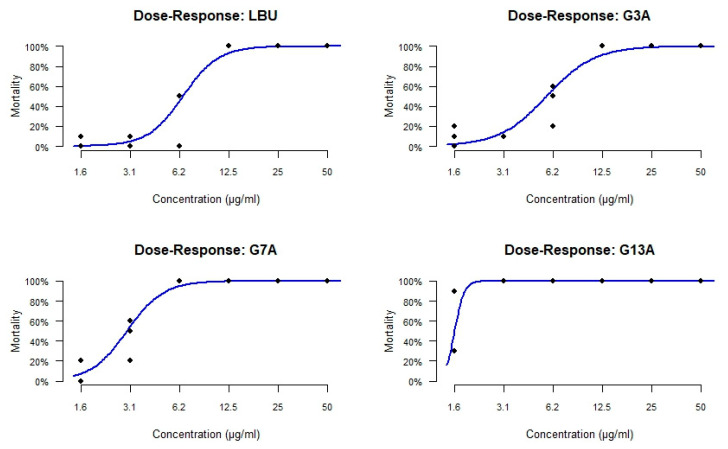
Dose–response mortality curves of *Danio rerio* embryos exposed to LBU and its Gly-to-Ala variants (G3A, G7A, G13A) at the 120th hour. Each curve represents the mortality percentage in response to increasing peptide concentrations (1.6 to 50 µg/mL), fitted using a nonlinear sigmoidal regression model.

The dose–response curves (Figure 6) confirmed these trends. LBU and G3A induced a more gradual increase in mortality with increasing concentration, whereas G7A and, especially, G13A produced steep mortality curves with high lethality, even at low concentrations. G13A displayed nearly complete mortality at concentrations ≥3.1 µg/mL, indicating a sharp toxic threshold.

TI values, calculated as the ratio of LC_50_ to MIC, were low across all peptides, indicating a narrow safety margin in the zebrafish model (Table 4). G3A exhibited the highest TI (0.91), suggesting a slightly better selectivity profile, followed by LBU (0.52) and G7A (0.48). G13A showed the lowest TI (0.25), reinforcing its poor therapeutic window due to high toxicity relative to its antimicrobial threshold.

**Figure 7:**
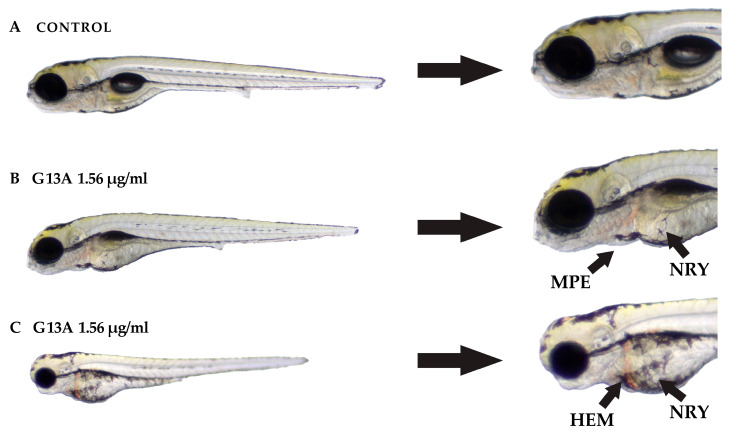
Representative *D. rerio* embryos following 120 h exposure to the G13A peptide at 1.56 µg/mL compared to an untreated control. (**A**) The control embryo displays normal morphology and development. (**B**,**C**) Embryos exposed to G13A exhibit multiple developmental abnormalities, including mild pericardial edema (MPE), hemorrhaging (HEM), and yolk sac retention (YSR). The embryo in (**C**) also shows general developmental delay. Arrows indicate regions of observed toxicity.

Developmental abnormalities were evaluated in *Danio rerio* embryos at 120 hpf following exposure to the tested peptides (Table S1). All peptides induced lethal egg coagulation at 12.5 and 25 µg/mL; however, G7A triggered coagulation already at 6.25 µg/mL, and G13A at concentrations as low as 1.56 µg/mL. Yolk sac retention was observed with LBU and G3A at 6.25 µg/mL, G7A at 3.13 µg/mL, and G13A at 1.56 µg/mL. Strikingly, only G13A caused hemorrhaging, general growth delay, and pericardial edema at 1.56 µg/mL, highlighting its significantly higher teratogenic potential compared to the other peptide variants. As shown in Figure 7, the control embryos developed normally without detectable malformations. In contrast, the embryos treated with G13A at 1.56 µg/mL exhibited dose-dependent developmental defects, including mild pericardial edema (MPE) and yolk sac retention (YSR), as well as more severe phenotypes, such as hemorrhage (HEM) and overall developmental delay. These findings suggest that even at low concentrations, G13A disrupts key processes in embryonic development.

These results suggest that single amino acid substitutions in the LBU peptide significantly alter its in vivo toxicity profile and development. In particular, the G13A variant markedly increased embryotoxicity in *D. rerio*, raising concerns about the safety of this modification despite potential improvements in antimicrobial potency.

### 2.4. Exploratory Gene Expression Profiling of LBU and Its Variants

Native LBU and the single-point variants G3A, G7A, and G13A were applied at sub-inhibitory concentrations (half MIC) to *S. aureus* ATCC 25923 grown in rich laboratory medium (LB) and in a host-mimicking fetal bovine serum (FBS).

**Figure 8:**
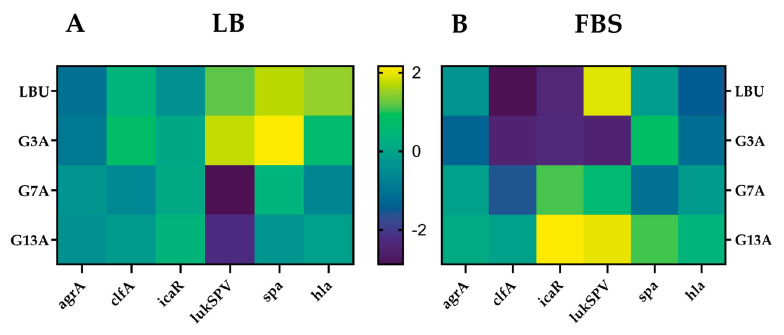
Heatmap visualization of virulence gene expression in *S. aureus* ATCC 25923 following treatments with LBU and its variants (G3A, G7A, G13A) under two different culture conditions. (**A**) Expression profiles in LB medium. (**B**) Expression profiles in fetal bovine serum (FBS). The color scale represents log_2_ fold changes relative to the untreated control, with purple indicating downregulation and yellow indicating upregulation.

Six virulence-related genes were selected (*agrA, clfA, icaR, lukSPV, spa*, and *hla*) to capture a representative profile of quorum sensing regulation (*agrA*), adhesion (*clfA*), biofilm repression (*icaR*), immune evasion (*spa*), and cytolytic toxin production (*lukSPV* and *hla*), providing an exploratory view of how peptide treatment influences the distinct profile of *S. aureus* pathogenicity. Transcript levels of six virulence-associated genes were quantified by RT-qPCR and expressed as log_2_ fold-change relative to the untreated control within each medium (Figure 8, Table S2).

LBU consistently suppressed *agrA* in LB medium (−1.10), whereas in FBS, it exhibited slight upregulation (0.39). The expression of *clfA* was mildly elevated in LB (0.37) but significantly downregulated in FBS (−1.89), indicating a reduction in fibronectin-binding capacity under host-like conditions. *IcaR* was modestly reduced in LB (−0.39) and strongly downregulated in FBS (−1.47), suggesting derepression of biofilm formation in serum. The leukocidin gene *lukSPV* was upregulated in both media (1.18 in LB; 2.46 in FBS), reflecting an increase in cytolytic toxin potential. *Spa* was highly expressed in LB (1.71) and remained moderately elevated in FBS (0.55), supporting an immune-evasive phenotype. Similarly, *hla* was strongly induced in LB (1.49), while it was downregulated in FBS (−0.61), indicating reduced hemolytic activity in serum.

G3A suppressed *agrA* expression in both LB (−0.93) and FBS (−0.47), demonstrating consistent but not biologically significant inhibition of quorum sensing. *clfA* was upregulated in LB (0.76) but significantly downregulated in FBS (−1.55), pointing to adhesion impairment under host-like conditions. *icaR* showed almost no change in LB (0.06) but was strongly repressed in FBS (−1.43). Notably, *lukSPV* was highly induced in LB (1.77) but significantly repressed in FBS (−1.54), highlighting a striking medium-dependent switch in cytotoxin regulation. *Spa* followed the same trend—upregulated in LB (2.16) and moderately elevated in FBS (1.43). *hla* expression was elevated in LB (0.60) but downregulated in FBS (−0.30), suggesting reduced cytolytic potential in serum.

G7A caused mild repression of *agrA* in LB (−0.32), but it was moderately upregulated in FBS (0.61), suggesting partial restoration of Agr activity under host-like conditions. *clfA* was downregulated in both media (−0.59 in LB, −0.69 in FBS), but neither met the significance cutoff. *IcaR* remained relatively stable in LB (0.10), while it was strongly upregulated in FBS (1.71), suggesting potential repression of biofilm formation. *lukSPV* was sharply repressed in LB (−2.88) but upregulated in FBS (1.22), underscoring medium-specific virulence modulation. *Spa* was mildly increased in LB (0.37) and slightly downregulated in FBS (−0.27). *Hla* was downregulated in LB (−0.62) and modestly upregulated in FBS (0.51), suggesting adaptation to serum conditions.

G13A showed mild repression of *agrA* in LB (−0.40) and moderate upregulation in FBS (0.82), implying potential restoration of quorum sensing. *clfA* was only slightly reduced in LB (−0.19) and moderately upregulated in FBS (0.64), contrasting with the other variants. *IcaR* was upregulated in LB (0.36) and strongly upregulated in FBS (2.66), implying inhibition of biofilm formation in serum. *LukSPV* was significantly repressed in LB (−2.28) and highly induced in FBS (2.49), reflecting a switch to a cytolytic, virulent phenotype under host-like conditions. *Spa* was downregulated in LB (−0.33) but strongly upregulated in FBS (1.69), indicating serum-enhanced immune evasion. Likewise, *hla* shifted from mild downregulation in LB (−0.11) to strong induction in FBS (1.04), confirming elevated hemolytic potential in serum.

Several peptides demonstrated striking medium-dependent regulatory shifts, with genes such as *lukSPV*, *icaR*, and *hla* showing opposite expression patterns between LB and FBS—indicative of a context-driven virulence modulation influenced by host-like conditions.

Further biological replicates and time-resolved analyses are required to validate these findings and assess their reproducibility and functional consequences.

## 3. Discussion

### 3.1. Antimicrobial Potency and Mechanistic Implications

The functional data can be rationalized in light of the predicted LBU structure. The in silico model projects a compact four-helix conformation of LBU. This is consistent with NMR studies of related class IId leaderless bacteriocins, which reveal a globular four-α-helix motif with a hydrophobic core and highly cationic surface [32,33]. In LBU, helix I (residues 2–12) and helix IV (residues 31–42) are predicted to be amphipathic and surface-exposed, making them prime candidates for membrane interaction. According to our previous study [18], 24 N-terminal amino acids are sufficient for antimicrobial activity, so our mutational analysis was concentrated on helix I. Furthermore, our previous study demonstrated that the synthetic form of LBU retains approximately 50% of the antimicrobial activity of the native LBU’s activity [18], so we have already shown that chemical synthesis is a feasible alternative for mutational analysis of LBU’s activity. The substitution of amino acids with alanine (called “alanine scan” [34]) is the most common way of inspecting the mutational effect since alanine’s tiny, inert methyl group preserves backbone conformation and a secondary structure bias, so any activity loss can be traced almost exclusively to the missing interactions of that residue’s side chain. It should be noted that a full alanine scan was not performed here; rather, the approach was selectively applied to investigate specific residue substitutions relevant to our study design. Replacing glycine residues in the N-terminal helix (helix I) with alanine (G3A, G7A) consistently enhanced anti-staphylococci activity, decreasing the MIC relative to LBU. This change suggests that increased helical propensity in helix I potentially strengthens bacterial membrane interactions, as it has been shown that Gly-to-Ala mutations stabilize helix integrity [35]. Notably, the G13A variant (adjacent to the central P14 kink) likewise showed a similar effect, indicating that reducing flexibility at the helix I-II junction may enhance the bacteriostatic effect, as it has been reported for the LV16 model helix [36]. In contrast, substitutions that disrupt hydrophobic or conformational features dramatically compromised LBU’s antimicrobial function [37]. The W2E variant, which replaces the predicted membrane-anchoring tryptophan with a negatively charged glutamate, diminished detectable activity, underscoring the potentially essential role of N-terminal hydrophobic/aromatic residues in inserting into bacterial membranes [38] or intramolecular interactions [39]. Similarly, disturbing the Pro14 hinge by alanine (P14A) or threonine (P14T) doubled the MIC and yielded incomplete killing (elevated minimum bactericidal concentration), highlighting that an intact helix–turn–helix motif might be crucial for optimal bactericidal action, as it has been shown for pore-forming peptides [40]. It is interesting that the importance of Pro-kink was reported in melittin [41], a main, α-helical component of bee venom (*Apis mellifera*). Apparently, in BHT-B bacteriocin, with an amino acid sequence virtually identical to LBU (see below), Thr is located at position 14. Thr can restore some backbone hydrogen bonding while retaining a bend, thereby modestly relaxing the hinge [42]. The effects of P14A and P14T variants on antimicrobial activity reflect the potential need for a rigid hinge at position 14 for full native LBU activity. Perhaps the most striking was the effect of histidine substitution at position 23: H23A (neutral alanine in place of positively charged histidine) completely switched off the antimicrobial activity. This indicates H23 is indispensable—likely serving a structural [43,44] or pH-dependent functional role [45]—while the moderate effect of H23R (His→Arg) substitution suggests that a permanent cation at this position can preserve the initial activity of LBU (MIC unchanged) but impairs bactericidal efficiency. Incidentally, when compared with the activity of BHT-B [46], which naturally contains T14 and N23, and the activity of BHT-B N23H variant (this study), these findings underscore the importance of the structural context of the entire peptide when designing and evaluating individual variants since introducing the same amino acid substitution into different peptide variants does not result in parallel changes in antimicrobial activity. Even the double LBU mutant, G3A/H23A, where the potent change is coupled with a hindering change, could not restore activity, emphasizing that position 23 is essential for the activity of LBU. Together, these findings illustrate that LBU’s potent activity stems from a delicate balance of helix stability, strategic flexibility, and charged/hydrophobic motif placement. Tightening [47] the helix I structure (via G→A) tends to enhance microbial membrane disruption, whereas removing key residues for membrane anchoring (W2) or dynamic insertion (P14, H23) tips the peptide toward inactivity. In mechanistic terms, helix I emerges as the key determinant of antimicrobial action, consistent with its amphipathic character and potential membrane disruption activity, while the central kink and interhelical loops enable the peptide to orient and deploy this and other helices effectively. The necessity of H23, which is predicted to participate in pH-dependent membrane disruption or dimerization, suggests that LBU might exploit environmental conditions (e.g., slightly acidic pH in infection sites [45] to maximize lethality. Loss of activity upon its removal (or loss of the imidazole protonation capability) points to H23 as a functional “trigger” residue, without which LBU cannot properly engage bacterial targets. Overall, the antimicrobial assays confirm that even single amino acid changes can profoundly alter LBU’s mechanism of action, either by enhancing membrane binding (as in Gly-to-Ala mutants) or by destabilizing critical structural interactions needed for bacterial killing. For future studies, more variants should be designed to fully inspect the mechanistic nature of LBU.

When placing these results in the broader context of bacteriocin research, similar mutational analyses have demonstrated that subtle changes can greatly impact activity, selectivity, and safety. For instance, alanine substitutions in plantaricin EF [48] similarly revealed position-dependent outcomes, where mutations at glycine residues in interhelical hinges drastically reduced antimicrobial activity and selectivity, mirroring our findings with G13A, but with increased antimicrobial activity. Likewise, Sakacin P [49] and Plantacyclin B21AG [50] demonstrated that single-residue substitutions, especially in positively charged or aromatic membrane-anchoring regions, significantly influenced their antimicrobial activity and specificity, consistent with our observations with the W2E and H23A variants. Investigations into lactococcin G and enterocin 1071 [51] also highlighted that changes at the peptide N-terminus profoundly affect strain specificity and potency, supporting our findings with G3A and G7A, where minor modifications substantially enhanced activity without compromising selectivity. Moreover, extensive protein engineering of nisin [52] revealed that conservative substitutions, such as glycine to alanine, could enhance antimicrobial activity by stabilizing helices involved in membrane interactions, while more disruptive mutations (e.g., negatively charged substitutions) severely reduced activity due to impaired membrane binding or altered peptide conformation, further supporting the mechanistic interpretations in our study. These comparative analyses underscore that the observed relationships between sequence modifications, structural integrity, and biological activity in LBU align well with established bacteriocin research, further validating our conclusions regarding critical residues and structural elements governing peptide functionality.

### 3.2. Cytotoxicity, Hemolysis, and In Vivo Safety

The safety profile of bacteriocins remains a major hurdle in their translation to therapeutic use. While they are generally considered safe at or near their MIC, their toxicity is often dose-dependent, and even some commercially available bacteriocins have been shown to exhibit adverse effects at higher concentration doses [53].

While certain mutations improved antimicrobial potency, our comprehensive safety assessment revealed that minor sequence changes can sharply shift LBU’s toxicity profile from benign to harmful. The synthetic LBU showed excellent selectivity regarding hemolysis and Caco-2 cytotoxicity. G3A and G7A preserved this safety profile. In contrast, G13A lost host selectivity, inducing hemolysis and Caco-2 cell death, which was reflected through low TI. This severe cytotoxicity indicates that the Gly13→Ala change converted LBU into a broad-spectrum membrane toxin that compromises mammalian cells almost as readily as bacteria. While G3A and G7A moderately increase helix I compactness and amphipathic character, G13A likely extends the hydrophobic face across the P14 pivot, yielding a peptide that can insert deeply and disrupt even cholesterol-rich eukaryotic membranes. Such a finding aligns with general observations that increased helix continuity and hydrophobicity correlate with higher hemolysis [54]. Glycine and proline in native AMPs often act as “safety valves” [54] that limit helix length or rigidity, thereby reducing lytic activity against host cells. Removing these residues can unleash a peptide’s latent cytotoxic potential—as we see with G13A transforming LBU into a toxin. The in vivo toxicity outcomes mirrored the in vitro trends, but all peptides displayed low TIs (<1), indicating general developmental toxicity. In the zebrafish embryo assays, G3A showed the highest TI, while G13A was markedly more lethal, with the lowest TI. Indeed, G13A caused nearly complete embryo mortality at lower doses, indicating a sharp toxicity. These data confirm that relatively conservative sequence changes can tilt LBU’s TI, in some cases drastically. The observed toxicity profiles of LBU variants have important implications for their potential clinical use. Both the parental peptide LBU and the variants G3A and G7A demonstrated relatively low hemolytic activity and cytotoxicity at concentrations near or above their antimicrobial MIC values, suggesting a favorable therapeutic window for these candidates. However, the G13A variant showed significantly increased hemolysis, cytotoxicity in Caco-2 cells, and pronounced embryotoxicity in the zebrafish model, resulting in a markedly narrower safety margin and poor therapeutic index. For therapeutic applications, LBU and G3A with higher therapeutic indices are more promising candidates due to their lower off-target toxicity, whereas variant G13A may pose unacceptable risks at effective antimicrobial doses. The narrow safety margins observed in vivo highlight the necessity of thorough preclinical toxicity evaluations, including multiple model systems, to ensure that peptides possess an acceptable risk–benefit profile. Ultimately, optimization efforts must prioritize improving selectivity to widen the therapeutic window, thus minimizing cytotoxic effects while retaining potent antimicrobial activity. From a safety standpoint, G13 emerges as a critical determinant of host compatibility: its presence in native LBU tempers the peptide’s aggressiveness, whereas its substitution triggers off-target lytic activity in cell and animal models. Importantly, the less deleterious nature of G3A and G7A suggests that not all helicity-enhancing mutations are equal—their position within the structure matters. Tweaking N-terminal flexibility (G3, G7) can boost antibacterial action without fully undermining selectivity, whereas perturbing the central kink (G13) crosses a threshold that leads to nonselective membrane disruption. While Caco-2 cells were used as a standard epithelial model to assess cytotoxicity, we acknowledge the limitation of relying on a single human cell line. However, this was complemented by the *Danio rerio* embryo assay, which provides a broader in vivo perspective, capturing systemic and developmental toxicity not reflected in in vitro models. Together, these approaches offer a multi-tiered view of peptide safety. Future studies will include additional mammalian cell lines relevant to therapeutic applications to further expand the toxicity profile. These findings underscore the need to evaluate toxicity early in AMP development: as demonstrated here, high antimicrobial potency often correlates with higher hemolysis and cytotoxicity [54,55], and only a fine line separates a beneficial peptide from a deleterious one. Any promising variant (like G7A or G3A) must therefore be vetted for safety in multiple models to ensure that enhanced microbicidal efficacy does not come at the cost of unacceptable host damage.

### 3.3. Virulence Modulation

Finally, with regard to virulence modulation, our exploratory gene expression profiling indicates that sub-inhibitory LBU and its variants can influence *S. aureus* regulatory pathways—a phenomenon increasingly recognized for AMPs [56,57,58,59]. At half MIC, the peptides are below bactericidal and bacteriostatic levels, creating an environment in which bacteria remain viable but sense membrane stress. Such doses are well known [58,60] to rewire staphylococcal systems such as Agr, SaeRS, GraSR, and σ^B. Fetal bovine serum (FBS) mimics the host milieu (high protein binding, iron limitation, complement) and often provokes virulence patterns that differ markedly from those seen in rich broth (LB) [60,61].

The *agrA* gene, a key regulator of *S. aureus* virulence [62], was uniformly downregulated by all the peptides in LB, consistent with AMP-mediated disruption of membrane integrity and quorum sensing [63,64]. However, the responses diverged in FBS: LBU, G7A, and G13A partially restored *agrA* expression—possibly overcoming serum-mediated suppression—whereas G3A strongly repressed it, suggesting distinct membrane interactions influenced by peptide structure. These *agrA* patterns shaped downstream targets: *clfA* (clumping factor A [65]) expression was inversely regulated, decreasing with *agrA* activation except in G13A in FBS, where both increased, hinting at regulatory decoupling via alternative systems (e.g., σ^B or MgrA [66]). Similarly, *icaR*, a biofilm repressor [67], was upregulated by G7A and G13A in both media, which was documented for AMP NH125 [57]. On the other hand, it was suppressed by LBU and G3A in FBS, potentially promoting biofilm formation under host-like conditions. The leucocidin [68] gene *lukS-PV* was repressed by G7A and G13A in LB but not in serum—suggesting that peptide efficacy against toxin expression is dampened by host components. *Spa* was broadly upregulated, enhancing immune evasion [69], except with G7A in FBS and G13A in LB, which showed downregulation and possible engagement of alternative regulation. Finally, *hla* was induced by LBU in LB, but by G7A and G13A only in FBS, pointing to serum-specific activation of cytotoxins. Collectively, these findings show that half-MIC doses of LBU and variants rewire the *S. aureus* virulence network in a context-specific manner, with structural variants yielding distinct regulatory outcomes depending on the growth environment. These findings are consistent with previously published data [61,70,71].

The observed gene expression changes can be rationalized through the structural properties of the peptide variants and their predicted membrane interactions. The G3A and G7A mutations, which replace glycine residues with alanine in helix I, likely strengthen membrane insertion and increase stress on the bacterial envelope, consistent with G3A’s strong downregulation of *clfA* and *icaR* in FBS and *lukSPV* repression under host-like conditions. These changes suggest more aggressive membrane engagement that triggers suppression of adhesion and toxin genes. In contrast, G13A, positioned near the P14 kink, may subtly alter interhelical angles without fully stabilizing the helix, which could explain its divergent effects: moderate *agrA* upregulation in FBS alongside strong induction of *icaR*, *spa*, and *hla*. This indicates a more dynamic interaction with bacterial membranes that modulates stress responses in a serum-dependent manner. LBU, with native flexibility, showed intermediate effects between these variants. Altogether, these findings highlight how small, localized changes in the helix structure and amphipathicity can reprogram bacterial virulence responses, supporting a model where structural tuning of antimicrobial peptides influences not just bactericidal action but also bacterial signaling and adaptation under host-like conditions.

### 3.4. Future Directions

Building on our current findings, future work will focus on several key areas to further develop LBU as a therapeutic candidate. This includes designing a broader mutational library to explore substitutions beyond helix I and the interhelical segment, with a focus on improving bacterial selectivity while minimizing cytotoxicity. Mechanistic studies using membrane permeability assays, circular dichroism spectroscopy, and molecular dynamics simulations are planned to validate membrane interaction hypotheses and refine structure–function models. In parallel, we aim to establish a recombinant expression system for LBU to enable scalable production and facilitate structure resolution studies via NMR or crystallography. Finally, preclinical validation in mammalian infection models will be undertaken to evaluate pharmacokinetics, safety, and in vivo efficacy of LBU and the most promising variants.

## 4. Materials and Methods

### 4.1. Peptides

Peptides were custom-synthesized by RoyoBiotech Co., Ltd. (Shanghai, China), using standard Fmoc solid-phase peptide synthesis techniques. The synthesized peptides were purified to >98% purity by reverse-phase high-performance liquid chromatography (RP-HPLC), and their identities were confirmed by electrospray ionization mass spectrometry (ESI-MS).

The peptides were supplied in lyophilized form and initially dissolved in sterile Milli-Q water. However, due to poor solubility, dissolution was repeated using 0.02 M HCl, a method commonly employed for solubilizing other cationic bacteriocins, such as nisin [72]. The acidic conditions improved peptide solubility without visible precipitation. The solutions were then pH-adjusted to 6.8 using 0.1 M Tris-HCl (pH 8.0), filter-sterilized (0.22 µm), aliquoted, and stored at −20 °C until use.

### 4.2. Structure Prediction

The three-dimensional structure of native LBU was obtained from the AlphaFold Protein Structure Database (https://alphafold.ebi.ac.uk/search/text/Bacteriocin%20lactolisterin%20BU?suggested=true; accessed on 13 June 2025). The predicted model was visualized in ChimeraX (version 1.9).

The hydrophobic moment (µH) and general hydrophobicity (H) of each LBU’s helices were quantified using the HeliQuest web server (https://heliquest.ipmc.cnrs.fr; accessed on 13 June 2025) [73], which calculates the mean hydrophobic dipole vector by applying the Eisenberg scale and an α-helix periodicity (100° per residue).

Polarity values were assessed using the ProtScale tool (https://web.expasy.org/protscale/) [74] based on the Zimmerman polarity scale, enabling visualization of polarity distribution along the peptide sequence.

### 4.3. Bacterial Strain and Culture Conditions

The bacterial strain used in this study was *S. aureus* ATCC 25923, obtained from the American Type Culture Collection (ATCC, Manassas, VA, USA). Bacteria were routinely cultured at 37 °C in either Luria–Bertani (LB) broth (10 g/L tryptone, 5 g/L yeast extract, 5 g/L NaCl) or fetal bovine serum (FBS; Gibco, Thermo Fisher Scientific) without additional supplementation. All experiments were initiated from overnight cultures grown under the same conditions. For all assays, bacterial suspensions were adjusted to a 0.5 McFarland standard (~1 × 10^8^ CFU/mL) using sterile 0.9% NaCl solution.

### 4.4. Antimicrobial Activity

#### 4.4.1. Broth Microdilution

MIC values were determined by broth microdilution following the guidelines of the European Committee on Antimicrobial Susceptibility Testing (EUCAST, Version 5.0), with a modification in the choice of medium. Instead of cation-adjusted Mueller–Hinton Broth (CA-MHB), Luria–Bertani (LB) broth was used for all MIC assays involving *S. aureus* ATCC 25923 to ensure consistency with conditions used in complementary experiments. Two-fold serial dilutions of peptides (ranging from 1.56 to 50 µg/mL) were prepared in sterile LB, inoculated with ~5 × 10^5^ CFU/mL bacterial suspension, and incubated at 37 °C for 18–20 h. MIC was defined as the lowest peptide concentration with no visible bacterial growth. All assays were performed in triplicate.

#### 4.4.2. Spot-on-Lawn Assay

The antimicrobial activity of LBU and its variants was initially screened using a spot-on-the-lawn assay, as reported by Kojic et al. [75]. *S. aureus* ATCC 25923 was grown overnight in LB broth, adjusted to 0.5 McFarland (~1 × 10^8^ CFU/mL), and mixed 1:100 into molten soft agar (0.7% agar in LB). Five milliliters of this suspension was overlaid onto LB agar plates and allowed to solidify.

Peptide stock solutions (1 mg/mL) were serially diluted in sterile water, and 5 µL of each dilution was carefully spotted onto the surface of the inoculated agar. The plates were dried under a laminar flow hood for 15–20 min and incubated overnight at 37 °C. Zones were observed, and their size and clarity were compared.

### 4.5. Hemolysis Assay

Hemolytic activity of peptides was assessed as described by Dong et al. [19], with some modifications. Fresh defibrinated sheep blood was obtained from the Institute of Virology, Vaccines and Sera “Torlak” (Belgrade, Serbia). Erythrocytes were washed three times with sterile Tris-buffered saline (TBS, 10 mM TRIS-HCl, pH 7.4, 150 mM NaCl) by centrifugation at 800× *g* for 5 min and resuspended to a final 2% (*v*/*v*) erythrocyte suspension in TBS.

Peptide solutions (0.02 M HCl) were diluted in Tris-buffered saline (TBS, 10 mM TRIS-HCl, pH 7.4, 150 mM NaCl) to final concentrations ranging from 6.25 to 50 µg/mL. In a 96-well flat-bottom plate, 100 µL of peptide solution was mixed with 100 µL of erythrocyte suspension and incubated at 37 °C for 1 h.

After incubation, the plates were centrifuged at 1000× *g* for 5 min, and 100 µL of the supernatant was transferred to a flat-bottom 96-well plate. Absorbance was measured at 540 nm using a microplate reader (model BioTek Epoch Microplate Spectrophotometer; Agilent Technologies, Winooski, VT, USA).

Hemolysis (%) was calculated relative to a negative control (TBS; 0% lysis) and a positive control (0.1% SDS; 100% lysis) using the formula:

$$\mathrm{Hemolysis}\;(\%)=\frac{A_{\mathrm{sample}}-A_{\mathrm{TBS}}}{A_{\mathrm{SDS}}-A_{\mathrm{TBS}}}\times 100$$

The assay was performed in triplicate.

### 4.6. In Vitro Caco-2 Toxicity

The cytotoxic effects of peptides were evaluated on human colorectal adenocarcinoma Caco-2 cells (ATCC HTB-37). The cells were cultured in Dulbecco’s Modified Eagle Medium (DMEM, high glucose; Gibco, Thermo Fisher Scientific) supplemented with 10% fetal bovine serum (FBS) and 1% penicillin–streptomycin. The cells were maintained at 37 °C in a humidified incubator with 5% CO_2_ and subcultured twice weekly, according to Popovic et al. [76].

Cytotoxic effects of peptides on Caco-2 cells were assessed using a lactate dehydrogenase (LDH) release assay according to the manufacturer’s protocol (Lactate Dehydrogenase Cytotoxicity Assay Kit (Thermo Fisher Scientific, Waltham, MA, USA)). The Caco-2 cells were seeded in 96-well flat-bottom plates at a density of 2 × 10^4^ cells per well and allowed to reach confluency over 48 h at 37 °C and 5% CO_2_.

After incubation, the cells were treated with peptides at final concentrations ranging from 3.125 to 50 µg/mL resuspended in DMEM. The cells were then incubated for 24 h. Untreated cells (incubated with medium only) served as a negative control, and cells treated with LDH Lysis buffer were used as a positive control.

Following peptide treatment, 50 µL of cell culture supernatant was transferred from each well into a new 96-well plate. LDH reaction mixture (50 µL) was added to each well and incubated at room temperature for 30 min in the dark. The absorbance was measured at 490 nm using a microplate reader.

Cytotoxicity (%) was calculated using the following formula:

$$\mathrm{Cytotoxicity}\;(\%)\;=\;\frac{A_{\mathrm{sample}}\;-\;A_{\mathrm{negative}}}{A_{\mathrm{positive}}\;-\;A_{\mathrm{negative}}}\;\times \;100$$

The assay was performed in triplicate.

### 4.7. In Vivo Danio Rerio Embryo Toxicity

The toxicity of synthetic peptide variants was assessed in vivo using Danio rerio embryos, in accordance with OECD Test Guideline 236 (Fish Embryo Acute Toxicity Test) and the European Directive 2010/63/EU on the protection of animals used for scientific purposes, according to Aleksic et al. [77]. All procedures were conducted at the Institute of Molecular Genetics and Genetic Engineering, University of Belgrade.

Wild-type adult zebrafish were maintained under standard laboratory conditions (28 °C; 14:10 h light–dark cycle). Embryos were obtained via pairwise mating and collected within 1 h post-fertilization (hpf). At 5 hpf, the embryos were randomly distributed into 24-well plates (10 embryos/well, 1 mL embryo medium per well) containing embryo water (0.2 mg/mL Instant Ocean^®^ salts in distilled water). Peptides were tested at five concentrations (e.g., 1.56–25 µg/mL) in triplicate. Peptide solvent was used as a negative control. The plates were incubated at 28 °C without media replacement.

Embryonic development was monitored using an inverted microscope (Zeiss Stemi 508; Carl Zeiss Microscopy GmbH, Jena, Germany). Lethal endpoints and sublethal malformations were scored according to OECD guidelines (Table S1). At 120 hpf, the embryos were anesthetized using 0.1% tricaine, photographed, and euthanized via freezing at −20 °C for 24 h.

LC_50_ values were estimated using the log–logistic binomial model (LL.2) from the drc package in R.

### 4.8. Virulence Gene Expression Analysis

The effect of sub-inhibitory peptide concentrations on *S. aureus* ATCC 25923 virulence gene expression was evaluated using quantitative real-time PCR (RT-qPCR). Bacteria were cultured overnight in LB broth and FBS at 37 °C and then diluted 1:100 into fresh LB and fetal bovine serum (FBS, Gibco), treated with peptides at half MIC concentrations for 16–18 h, and incubated at 37 °C with 180 rpm shaking. Untreated bacterial cultures in corresponding media served as controls.

Total RNA was extracted using the RNeasy Mini Kit (QIAGEN GmbH, Hilden, Germany). Samples were treated with Dnase I (Thermo Fisher Scientific) to remove genomic DNA contamination. RNA concentration and purity were determined using a NanoDrop spectrophotometer (A_260_/A_280_ ratio 1.9–2.1).

Complementary DNA (cDNA) synthesis was performed using 200 µg of total RNA and the RevertAid RT Reverse Transcription Kit (Thermo Fisher Scientific) with random hexamer primers, according to the manufacturer’s instructions. Quantitative PCR was performed using FastGen 2x IC Green qPCR Universal (Nippon Genetics) on a LineGene 9600 Plus Real Time thermocycler (Bioer Technology Co., Ltd., Hangzhou, China). Primer sequences (Table S3) for the virulence-related genes *agrA*, *clfA*, *hla*, *icaR*, *lukS-PV*, and *spa* were used. The housekeeping gene *gmk* (guanylate kinase) was used for normalization.

Primer efficiency (90–110%) and specificity were confirmed by melt curve analysis. Relative expression levels were calculated using the ΔΔCt method, as reported by Livak et Schmittgen [78], comparing treated samples to their corresponding untreated controls in each medium. A log_2_ fold change ≥1 or ≤−1 was considered biologically significant. All experiments were performed in duplicate.

### 4.9. Statistical Analysis

All statistical analyses were performed using GraphPad Prism (version 9.0.0; GraphPad Software, San Diego, CA, USA). Data were expressed as mean ± standard deviation (SD). Graphs were generated and annotated in GraphPad Prism.

Dose–response curves and LC_50_ values were estimated using the log–logistic binomial model (LL.2) from the drc package in R [79] (version 2025.05.0). Mortality data were modeled as proportions of dead individuals relative to total individuals tested, and 95% confidence intervals for LC_50_ were calculated using the delta method.

## 5. Conclusions

This study emphasizes the critical need to evaluate all functional consequences of amino acid modifications. While certain variants may exhibit improved antimicrobial potency, their therapeutic application must be carefully weighed against their safety profiles. Our findings suggest that natural bacteriocins like LBU are evolutionarily optimized to balance antimicrobial activity with host specificity and minimal off-target toxicity. The results presented here advance the understanding of LBU’s structure–function relationship and identify Gly-to-Ala substitutions in amphipathic helices as rational modifications for activity enhancement—though not without toxicological trade-offs.

Moreover, the multidisciplinary approach employed here—integrating in silico modeling, mutational screening, cytotoxicity assays in cell lines and embryos, and virulence gene modulation—provides a comprehensive template for the preclinical evaluation of next-generation bacteriocins.

## Figures and Tables

**Table 1:** Overview of LBU peptide variants. Variants are grouped based on the hypothesized role of targeted residues in structural stability or biological activity.

Group	Variant Name	Variation	Region
I helix stability	G3A	Gly3 → Ala	N-terminus of I helix
	G7A	Gly7 → Ala	Core of I helix
	W2E	Try2 → Glu	N-terminus of I helix
Proline role	G13A	Gly13 → Ala	Adjacent to proline kink
	P14T	Pro14 → Thr	Proline kink
	P14A	Pro14 → Ala	Proline kink
Histidine role	H23A	His23 → Ala	Histidine
	H23R	His23 → Arg	Histidine
Double mutant	G3A/H23A	Gly3 and H23 → Ala	I helix and histidine
BHT-B	BHT-B	Original sequence	-
	BHT-B_N23H	Asn23 → His	Asparagine

**Table 2:** MIC and MBC of the parental peptide LBU and its amino acid variants against *S. aureus* ATCC 25923.

Peptide	MIC (µg/mL)	MBC (µg/mL)
LBU	12.5	25
W2E	>50	>50
G3A	6.25	25
G7A	6.25	25
G13A	6.25	12.5
P14A	25	>50
P14T	25	>50
H23R	12.5	50
H23A	>50	>50
G3A_H23A	>50	>50
BHT-B	12.5	25
BHT-B_N23H	50	>50

**Table 3:** LC_50_ and TI of LBU and its Gly-to-Ala variants (G3A, G7A, G13A) based on cytotoxic activity on Caco-2 cells at increasing peptide concentrations. Data are presented as mean ± standard deviation (*n* = 3). TI is the ratio of LC_50_/MIC.

Peptide	LC_50_	MIC (µg/mL)	TI
LBU	>50	12.5	>4
G3A	43.91 ± 2.47	6.25	7.03
G7A	31.93 ± 1.07	6.25	5.11
G13A	7.49 ± 0.26	6.25	1.20

**Table 4:** Developmental toxicity of LBU and its Gly-to-Ala variants (G3A, G7A, G13A) on Caco-2 cells at increasing peptide concentrations. Data are presented as mean ± standard deviation (*n* = 3). TI is the ratio of LC_50_/MIC.

Peptide	LC_50_	MIC (µg/mL)	TI
LBU	6.54 ± 0.49	12.5	0.52
G3A	5.69 ± 0.5	6.25	0.91
G7A	2.98 ± 0.23	6.25	0.48
G13A	1.56 ± 0.04	6.25	0.25

## Data Availability

The dataset is available on request from the authors.

## Supplementary Materials

The following supporting information can be downloaded at: https://www.mdpi.com/article/10.3390/molecules30153134/s1, Table S1: Observed developmental abnormalities in *D. rerio* embryos following exposure to peptides LBU and its Gly-to-Ala variants across five concentrations after 120 hpf; Table S2: Log_2_ fold changes in virulence gene expression of *S. aureus* ATCC 25923 treated with LBU and its Gly-to-Ala variants under different media conditions; Table S3: Primer sequences used for quantitative real-time PCR (qPCR) analysis of *Staphylococcus aureus* genes.
