# Supplementary material for: Amino Acid Substitutions in Bacteriocin Lactolisterin BU Reveal Functional Domains Involved in Biological Activity Against Staphylococcus aureus
Source: Molecules. 2025 Jul 26;30(15):3134. doi: 10.3390/molecules30153134 (PMC12348470; doi:10.3390/molecules30153134)
Supplement: Supplementary file 1 [file molecules-30-03134-s001.zip › molecules-3755953-supplementary.pdf]

Table S1. Observed developmental abnormalities in *D. rerio* embryos following exposure to peptides LBU and its Gly-to-Ala variants across five concentrations after 120 hpf. The endpoints are grouped into three categories: lethal effects (e.g., coagulated eggs, non-detachment of the tail), teratogenic effects (e.g., malformations, growth retardation, yolk sac retention), and cardiotoxicity (pericardial edema). Presence of a deformity under a given treatment condition is indicated by a checkmark (●). Blank cells denote absence of observable effect under the corresponding condition.

		Peptides																			
		LBU					G3A					G7A					G13A				
	Concentration (µg/ml)	1.56	3.13	6.25	12.5	25	1.56	3.13	6.25	12.5	25	1.56	3.13	6.25	12.5	25	1.56	3.13	6.25	12.5	25
Category	Developmental endpoint																				
Lethal effect	Coagulated eggs				●	●				●	●			●	●	●		●	●	●	●
	Lack of somite formation																				
	Non-detachment of the tail																				
	Lack of the heart beating																				
Teratogenic effect	Malformation of head																				
	Malformation of eyes																				
	Malformation of sacculi/otoliths																				
	Malformation of chorda																				
	Malformation of tail																				
	Scoliosis																				
	Yolk edema																				
	Yolk sac retention			●					●				●				●				
	Growth retardation																●				
	Hatching																				
	Hemorrhage																●				
Cardiotoxicity	Pericardial edema																●				

Table S2. Log_2 fold changes in virulence gene expression of *S. aureus* ATCC 25923 treated with LBU and its Gly-to-Ala variants under different media conditions.

Medium	Peptides	Genes					
		agrA	*clfA*	*icaR*	*lukSPV*	*spa*	*hla*
LB	LBU	-1.10	0.37	-0.39	1.18	1.71	1.49
	G3A	-0.93	0.76	0.06	1.77	2.16	0.60
	G7A	-0.32	-0.59	0.10	-2.88	0.37	-0.62
	G13A	-0.40	-0.19	0.36	-2.28	-0.32	-0.11
FBS	LBU	0.39	-1.89	-1.47	2.46	0.55	-0.61
	G3A	-0.47	-1.55	-1.43	-1.54	1.43	-0.30
	G7A	0.61	-0.69	1.71	1.22	-0.27	0.51
	G13A	0.82	0.64	2.66	2.49	1.69	1.04

Table S3. Primer sequences used for quantitative real-time PCR (qPCR) analysis of *Staphylococcus aureus* genes. For each gene, forward (Fw) and reverse (Rev) primer sequences are listed along with the expected amplicon length. All primers were designed in this study.

Gene	Primer name	Sequence (5′-3′)	Length (bp)	Source
agrA	*agrA*_Fw	ATGGTATCAAATTAGGCAG	171	This study
	*agrA*_Rev	AACAGTCTATAATTCGAGT		
clfA	clfA_Fw	GTAATGAAACGACTTCTAAT	164	This study
	clfA_Rev	ATCTTTATTACTTGCATC		
icaR	*icaR*_Fw	ATCGAACTATTCAATTGATG	174	This study
	*icaR*_Rev	TTTTGCTATCTCTTTACT		
luxSPV	*luxSPV*_Fw	AGAAACAGTTGCAATAAATGAAGG	99	This study
	*luxSPV*_Rev	CCATATAGTCAAAATCCGAGAGAC		
spa	*spa*_Fw	GCGCCTTCTATGAAATCTTGAA	140	This study
	*spa*_Rev	CGGTGCTTGAGATTCGTTTAA		
hlA	hlA_Fw	TACAGATATTGGAAGCAA	165	This study
	hlA_Rev	CTATATTGACCAGCAATGG		
gmk	gmk_Fw	TGGATAATGAAAAAGGATTG	162	This study
	gmk_Rev	ATCTACGCCATCAACTTCAC		
